# 1-Isopropyl­ideneamino-1*H*-tetra­zol-5-amine

**DOI:** 10.1107/S1600536809024994

**Published:** 2009-07-04

**Authors:** Chun-Lin He, Zhi-Ming Du, Zheng-Qiang Tang, Xiao-Min Cong, Ling-Qiao Meng

**Affiliations:** a State Key Laboratory of Explosion Science and Technology, Beijing Institute of Technology, Beijing 100081, People’s Republic of China

## Abstract

The mol­ecule of the title compound, C_4_H_8_N_6_, assumes an approximately planar structure, the methyl C atoms and the C atom to which they are bonded being out of the mean tetrazole ring plane by 0.108 and 0.139, and 0.144 Å, respectively. π–π stacking between parallel tetra­zole rings [centroid–centroid distance = 3.4663 (11) Å] is observed in the crystal structure. Inter­molecular N—H⋯N hydrogen bonding further helps to stabilize the crystal structure.

## Related literature

For the preparation of the title compound, see: Gaponnik & Karavai (1984 ▶). For general background, see: Galvez-Ruiz *et al.* (2005 ▶); Joo *et al.* (2008 ▶). For a related structures, see: Lyakhov *et al.* (2005 ▶).
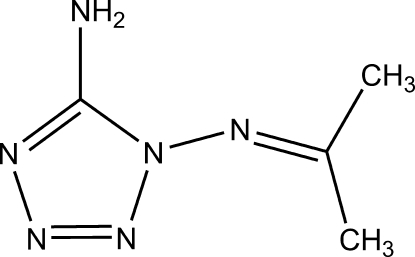


## Experimental

### 

#### Crystal data

C_4_H_8_N_6_
*M* *_r_* = 140.16
Monoclinic, 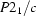

*a* = 7.488 (2) Å
*b* = 7.4238 (19) Å
*c* = 11.997 (3) Å
β = 97.145 (3)°
*V* = 661.7 (3) Å^3^
*Z* = 4
Mo *K*α radiation
μ = 0.10 mm^−1^
*T* = 93 K
0.43 × 0.43 × 0.33 mm

#### Data collection

Rigaku Saturn724+ diffractometer
Absorption correction: none
5172 measured reflections
1520 independent reflections
1334 reflections with *I* > 2σ(*I*)
*R*_int_ = 0.024

#### Refinement

*R*[*F* ^2^ > 2σ(*F* ^2^)] = 0.036
*wR*(*F* ^2^) = 0.088
*S* = 1.00
1520 reflections
101 parameters
H atoms treated by a mixture of independent and constrained refinement
Δρ_max_ = 0.28 e Å^−3^
Δρ_min_ = −0.17 e Å^−3^

### 

Data collection: *CrystalClear* (Rigaku, 2008 ▶); cell refinement: *CrystalClear*; data reduction: *CrystalClear*; program(s) used to solve structure: *SHELXS97* (Sheldrick, 2008 ▶); program(s) used to refine structure: *SHELXL97* (Sheldrick, 2008 ▶); molecular graphics: *ORTEP-3* (Farrugia, 1997 ▶); software used to prepare material for publication: *WinGX* (Farrugia, 1999 ▶).

## Supplementary Material

Crystal structure: contains datablocks global, I. DOI: 10.1107/S1600536809024994/xu2546sup1.cif

Structure factors: contains datablocks I. DOI: 10.1107/S1600536809024994/xu2546Isup2.hkl

Additional supplementary materials:  crystallographic information; 3D view; checkCIF report

## Figures and Tables

**Table 1:** Hydrogen-bond geometry (Å, °)

*D*—H⋯*A*	*D*—H	H⋯*A*	*D*⋯*A*	*D*—H⋯*A*
N6—H6*A*⋯N2^i^	0.890 (16)	2.200 (17)	3.0600 (16)	162.6 (13)
N6—H6*B*⋯N1^ii^	0.876 (15)	2.118 (15)	2.9770 (16)	166.4 (14)

Symmetry codes: (i) 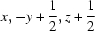; (ii) 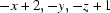.

**Fig. 1.**
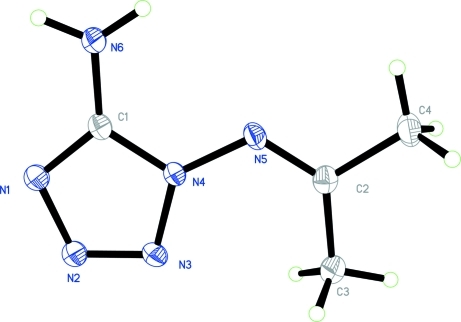
The molecular structure of the title compound with the atom-numbering scheme. Displacement ellipsoids are drawn at the 50% probability level.

## supplementary crystallographic information

### Comment

1,5-diaminotetrazole and its derivatives have received an increasing interest as a class of nitrogen-rich energetic materials during the last years.These compounds exhibit prospective application in generation of gases, propellants and other combustible and thermally decomposing systems (Galvez-Ruiz *et al.*, 2005; Joo,*et al.* 2008). The title compound had been prepared by Gaponnik & Karavai, but its crystal structure hadn't been reported, therefore, the structure of the title compound has been determined in our present work.

The crystal structure of the title compound is presented in Fig. 1, The bond distances and bond angles in the title compound are similar to the corresponding distances and angles reported by Lyakhov *et al.* (2005). The molecule is almost planar with only C2, C3 and C4 being out of the mean plane of the tetrazole ring by 0.108, 0.144 and 0.139 Å, respectively.

In the crystal structure the molecules are linked to each other via N—H···N hydrogen bonding (Table 1), forming a three dimensional network structure). The offset face-to-face π-π contact between the tetrazole rings, related by an inversion center, further helps to stabilize the crystal structure; centroid-centroid distance being 3.4663 (11) Å.

### Experimental

The title compound was prepared according to the literature method (Gaponnik & Karavai, 1984). The purity of the compound was checked by determining its melting point, m.p. 445–446 K. Crystals suitable for X-ray structure determination were obtained by slow evaporation of an acetone solution at room temperature.

### Refinement

Amino H atoms were located in a difference Fourier maps and were refined isotropically. Other H-atoms were placed in calculated positions with C—H = 0.98 Å, and refined in riding mode with U_iso_ = 1.2U_eq_(C).

### Crystal data

**Table:**

C_4_H_8_N_6_	*F*(000) = 296
*M**_r_* = 140.16	*D*_x_ = 1.407 Mg m^−^^3^
Monoclinic, *P*2_1_/*c*	Melting point: 445 K
Hall symbol: -P 2ybc	Mo *K*α radiation, λ = 0.71073 Å
*a* = 7.488 (2) Å	Cell parameters from 2076 reflections
*b* = 7.4238 (19) Å	θ = 3.1–27.5°
*c* = 11.997 (3) Å	µ = 0.10 mm^−^^1^
β = 97.145 (3)°	*T* = 93 K
*V* = 661.7 (3) Å^3^	Block, colourless
*Z* = 4	0.43 × 0.43 × 0.33 mm

### Data collection

**Table:**

Rigaku Saturn724+ diffractometer	1334 reflections with *I* > 2σ(*I*)
Radiation source: Rotating Anode	*R*_int_ = 0.024
graphite	θ_max_ = 27.5°, θ_min_ = 3.2°
Detector resolution: 28.5714 pixels mm^-1^	*h* = −9→9
multi–scan	*k* = −8→9
5172 measured reflections	*l* = −15→15
1520 independent reflections	

### Refinement

**Table:**

Refinement on *F*^2^	0 restraints
Least-squares matrix: full	H atoms treated by a mixture of independent and constrained refinement
*R*[*F*^2^ > 2σ(*F*^2^)] = 0.036	*w* = 1/[σ^2^(*F*_o_^2^) + (0.045*P*)^2^ + 0.16*P*] where *P* = (*F*_o_^2^ + 2*F*_c_^2^)/3
*wR*(*F*^2^) = 0.088	(Δ/σ)_max_ < 0.001
*S* = 1.00	Δρ_max_ = 0.28 e Å^−^^3^
1520 reflections	Δρ_min_ = −0.17 e Å^−^^3^
101 parameters	

### Special details

**Table:**

Geometry. All e.s.d.'s (except the e.s.d. in the dihedral angle between two l.s. planes) are estimated using the full covariance matrix. The cell e.s.d.'s are taken into account individually in the estimation of e.s.d.'s in distances, angles and torsion angles; correlations between e.s.d.'s in cell parameters are only used when they are defined by crystal symmetry. An approximate (isotropic) treatment of cell e.s.d.'s is used for estimating e.s.d.'s involving l.s. planes.
Refinement. Refinement of *F*^2^ against ALL reflections. The weighted *R*-factor *wR* and goodness of fit *S* are based on *F*^2^, conventional *R*-factors *R* are based on *F*, with *F* set to zero for negative *F*^2^. The threshold expression of *F*^2^ > σ(*F*^2^) is used only for calculating *R*-factors(gt) *etc*. and is not relevant to the choice of reflections for refinement. *R*-factors based on *F*^2^ are statistically about twice as large as those based on *F*, and *R*- factors based on ALL data will be even larger.

### Fractional atomic coordinates and isotropic or equivalent isotropic displacement parameters (Å^2^)

**Table:**

	*x*	*y*	*z*	*U*_iso_*/*U*_eq_	
N1	0.90734 (13)	0.19832 (13)	0.44509 (8)	0.0177 (2)	
N2	0.84796 (13)	0.34792 (13)	0.38501 (8)	0.0182 (2)	
N3	0.78344 (13)	0.46815 (13)	0.44617 (8)	0.0181 (2)	
N4	0.79984 (12)	0.39605 (12)	0.55284 (7)	0.0144 (2)	
N5	0.74880 (12)	0.46344 (12)	0.65193 (7)	0.0165 (2)	
N6	0.91309 (15)	0.12338 (14)	0.63884 (8)	0.0223 (2)	
C1	0.87689 (14)	0.23122 (15)	0.55029 (9)	0.0153 (2)	
C2	0.66745 (14)	0.61599 (15)	0.65364 (9)	0.0169 (2)	
C3	0.61800 (16)	0.74455 (16)	0.55860 (10)	0.0215 (3)	
H3A	0.7275	0.7992	0.5366	0.026*	
H3B	0.5397	0.8391	0.5825	0.026*	
H3C	0.5545	0.6795	0.4945	0.026*	
C4	0.62021 (16)	0.66948 (17)	0.76627 (10)	0.0232 (3)	
H4A	0.6608	0.5760	0.8213	0.028*	
H4B	0.4895	0.6838	0.7624	0.028*	
H4C	0.6794	0.7837	0.7891	0.028*	
H6A	0.887 (2)	0.156 (2)	0.7063 (14)	0.033 (4)*	
H6B	0.972 (2)	0.025 (2)	0.6261 (13)	0.037 (4)*	

### Atomic displacement parameters (Å^2^)

**Table:**

	*U*^11^	*U*^22^	*U*^33^	*U*^12^	*U*^13^	*U*^23^
N1	0.0210 (5)	0.0176 (5)	0.0146 (5)	0.0012 (4)	0.0026 (4)	−0.0006 (4)
N2	0.0214 (5)	0.0174 (5)	0.0161 (5)	0.0008 (4)	0.0036 (4)	−0.0003 (4)
N3	0.0230 (5)	0.0186 (5)	0.0133 (4)	0.0000 (4)	0.0045 (4)	0.0006 (4)
N4	0.0175 (5)	0.0144 (5)	0.0117 (4)	0.0008 (3)	0.0030 (3)	−0.0010 (3)
N5	0.0192 (5)	0.0183 (5)	0.0127 (4)	−0.0001 (4)	0.0042 (4)	−0.0031 (4)
N6	0.0337 (6)	0.0202 (5)	0.0137 (5)	0.0093 (4)	0.0062 (4)	0.0008 (4)
C1	0.0152 (5)	0.0167 (5)	0.0141 (5)	−0.0007 (4)	0.0026 (4)	−0.0019 (4)
C2	0.0151 (5)	0.0167 (5)	0.0190 (6)	−0.0019 (4)	0.0025 (4)	−0.0029 (4)
C3	0.0243 (6)	0.0185 (6)	0.0220 (6)	0.0043 (4)	0.0045 (5)	0.0002 (5)
C4	0.0261 (6)	0.0244 (6)	0.0196 (6)	0.0046 (5)	0.0043 (5)	−0.0055 (5)

### Geometric parameters (Å, °)

**Table:**

N1—C1	1.3326 (14)	N6—H6B	0.878 (17)
N1—N2	1.3678 (13)	C2—C4	1.4924 (16)
N2—N3	1.2870 (13)	C2—C3	1.4977 (16)
N3—N4	1.3784 (13)	C3—H3A	0.9800
N4—C1	1.3549 (14)	C3—H3B	0.9800
N4—N5	1.3866 (12)	C3—H3C	0.9800
N5—C2	1.2873 (15)	C4—H4A	0.9800
N6—C1	1.3309 (14)	C4—H4B	0.9800
N6—H6A	0.891 (17)	C4—H4C	0.9800
			
C1—N1—N2	105.52 (9)	N5—C2—C3	128.40 (10)
N3—N2—N1	112.53 (9)	C4—C2—C3	117.11 (10)
N2—N3—N4	105.28 (9)	C2—C3—H3A	109.5
C1—N4—N3	108.59 (9)	C2—C3—H3B	109.5
C1—N4—N5	120.58 (9)	H3A—C3—H3B	109.5
N3—N4—N5	130.82 (9)	C2—C3—H3C	109.5
C2—N5—N4	120.83 (9)	H3A—C3—H3C	109.5
C1—N6—H6A	121.1 (10)	H3B—C3—H3C	109.5
C1—N6—H6B	114.8 (10)	C2—C4—H4A	109.5
H6A—N6—H6B	123.9 (14)	C2—C4—H4B	109.5
N6—C1—N1	127.15 (11)	H4A—C4—H4B	109.5
N6—C1—N4	124.77 (10)	C2—C4—H4C	109.5
N1—C1—N4	108.08 (9)	H4A—C4—H4C	109.5
N5—C2—C4	114.48 (10)	H4B—C4—H4C	109.5
			
C1—N1—N2—N3	−0.23 (12)	N2—N1—C1—N4	0.45 (11)
N1—N2—N3—N4	−0.08 (12)	N3—N4—C1—N6	179.91 (10)
N2—N3—N4—C1	0.37 (11)	N5—N4—C1—N6	−1.31 (16)
N2—N3—N4—N5	−178.25 (10)	N3—N4—C1—N1	−0.52 (12)
C1—N4—N5—C2	−176.51 (10)	N5—N4—C1—N1	178.26 (9)
N3—N4—N5—C2	1.97 (17)	N4—N5—C2—C4	179.59 (9)
N2—N1—C1—N6	−179.99 (11)	N4—N5—C2—C3	−1.35 (17)

### Hydrogen-bond geometry (Å, °)

**Table:**

*D*—H···*A*	*D*—H	H···*A*	*D*···*A*	*D*—H···*A*
N6—H6A···N2^i^	0.890 (16)	2.200 (17)	3.0600 (16)	162.6 (13)
N6—H6B···N1^ii^	0.876 (15)	2.118 (15)	2.9770 (16)	166.4 (14)

Symmetry codes: (i) *x*, −*y*+1/2, *z*+1/2; (ii) −*x*+2, −*y*, −*z*+1.
